# The botanical education extinction and the fall of plant awareness

**DOI:** 10.1002/ece3.9019

**Published:** 2022-07-10

**Authors:** Sebastian Stroud, Mark Fennell, Jonathan Mitchley, Susannah Lydon, Julie Peacock, Karen L. Bacon

**Affiliations:** ^1^ School of Biology, Faculty of Biological Sciences University of Leeds Leeds UK; ^2^ AECOM Cambridge UK; ^3^ School of Biological Sciences University of Reading Reading UK; ^4^ School of Biosciences University of Nottingham Loughborough UK; ^5^ School of Geography, Faculty of Environment University of Leeds Leeds UK; ^6^ Botany and Plant Sciences, Ryan Institute, School of Natural Sciences National University of Ireland Galway Ireland

## Abstract

Civilization is dependent upon plants for survival. Plants permeate our every moment and our relationship with them will dictate how we will manage the threats of climate change and ecological collapse defining the Anthropocene. Yet, despite the significance of plants and the critical role they have played in shaping ecosystems, civilizations, and human cultures, many people are now disconnected from the botanical world. Students are presented with little plant content, particularly identification, compared with animal content. Consequently, we are producing few plant scientists and educating fewer scientists about plants. This drives a self‐accelerating cycle we term *the extinction of botanical education*. A process of knowledge erosion, that in this instance contributes to our separation from the natural world, makes us blind to the biodiversity crisis and inhibits our ability to restore it. We argue that neglecting the importance of plants within education threatens the foundations of industries and professions that rely on this knowledge. Furthermore, this extinction of botanical education creates an existential threat: Without the skills to fully comprehend the scale of and solutions to human‐induced global change, how do we as a society combat it? We present key research agendas that will enable us to reverse the extinction of botanical education and highlight the critical role plants play on the global stage.

## Graphical abstract

Civilization is dependent upon plants for survival and our relationship with them will dictate how we will manage the global impact of humanity which defines the Anthropocene. We document and define a self‐perpetuating educational cycle that we term the extinction of botanical education and its impact on the science of botany and potential ramifications for society to reverse and stabilise human included global change.

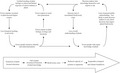

## 1 THE KNOWN PROBLEM

We are entering the most critical period of change within human history and facing our largest existential threats to survival (Ruckelshaus et al., 2020; Skea et al., 2021). The unprecedented rate of expansion, consumption, and ecological destruction since the Industrial Revolution and during the Anthropocene (Steffen, Broadgate, et al., 2015) has pushed our climate and ecosystems closer to irreversible tipping points (Steffen, Richardson, et al., 2015), beyond which their capacity to support human civilization is uncertain (Ruckelshaus et al., 2020). From our earliest settlements, we have been reliant on plants to provide shelter, fuels, and sustenance and are the basis of nearly all terrestrial ecosystems (Lev‐Yadun et al., 2000). Plants perform a huge variety of essential functions, from forming soils to producing oxygen, yet their neglect is costing us dearly (Bardgett et al., 2021). From birth to death, plants dominate every aspect of our existence—they are our silent partners in civilization (Adamo et al., 2021). There is virtually always a plant or a plant product in your field of vision. Despite this significance, plants are currently underappreciated and neglected in many sectors of modern society, and there has been little research into identifying the measurable consequences of our plant apathy toward societies' challenges (Amprazis & Papadopoulou, 2020; Jose et al., 2019). Globally, the picture is worrying: ~40% of plant species are threatened with extinction (Nic Lughadha et al., 2020). Human society is reliant on plants for the maintenance of our biosphere and life systems yet continued human activities place immense pressure on ecosystems and species.

We pose that a botanist had a wide breadth of skills to draw upon. This is beyond the ability to identify plants and recall specific species ecological knowledge but a broad spectrum of understanding from plant physiology, identification, taxonomy, ecology, genetic, and cellular plant biology. Botanists are trained not only in understanding plants from form to function and ecology to physiology but beyond their processes and function, to their social and cultural contexts.

Solutions and pathways to a sustainable future are directly linked to our understanding of plants and the processes and services they provide and facilitate (UN DESA, 2016). Plants will play a key role in achieving many of the United Nations (UN) Sustainable Development Goals (SDGs). Plants are fundamental to SDG 15, Life on land. Plants by biomass comprise the majority of the living organisms on Earth (Bar‐On et al., 2018), they provide the food source to nearly every single ecosystem. This is obvious regarding terrestrial ecosystems, but plants also play an essential role underwater as algae and phytoplankton. For example, plants can trap and convert radiation into renewable fuels, which may help the UN to meet several SDGs including Goal 7 (Affordable and Clean Energy) and Goal 12 (Responsible Consumption and Production) (Amprazis & Papadopoulou, 2020; UN DESA, 2016). Further examinations reveal the utilization of plants relates directly to meeting all seventeen of the UN's SDGs (Amprazis & Papadopoulou, 2020), yet specific plant conservation objectives, such as the Global Strategy for Plant Conservation (Jackson & Kennedy, 2009), have been poorly integrated into national biodiversity policies. Other objectives may lack a specific botanical focus or expertise (Sharrock & Jackson, 2017).

In the face of global change, there is no doubt that restoration of habitat and utilization of processes that plants mediate will be central to combating the looming climate and ecological crises of the 21st century (Queiroz et al., 2021). We ignore the opportunities presented to us by the botanical world at our peril.

Here, we highlight how the lack of plant awareness and the continued lack of detailed inclusion of plants in education have exacerbated this crisis. This situation drives us ever closer to losing our ability to build a sustainable and ecologically robust future.

### 1.1 The state of botanical teaching in the United Kingdom

Given the critical importance of plants in addressing the challenges facing humanity today, is the role that botany plays in addressing global change recognized? There has been a call to action within the science to “engage the power of the public with the power of plants” (Crane et al., 2019). However, botany, once a compulsory component of many biology degrees and school programmes, is now practically nonexistent in the United Kingdom. Instead, the programmes offered are Plant Science or Plant Biology, and these are offered by only 11 universities (Table A1). The United Kingdom has recently announced the creation of a Natural science GSCE (Gov.uk, 2022). This could provide more plant identification and ecology in formal schooling; however, it is unclear yet as to what the focus of the curriculum will be, although flora and fauna are listed first in popular content themes (Oates & Duffy, 2020). Depending on the content and format, there is again the risk of reducing plants to processes. There is additionally the question of who will teach it. Many teachers may have little experience to teach plant identification and ecology.

We argue that these plant‐specific degrees are too few and current general biology programmes may not offer the broad spectrum of plant knowledge that is needed in light of progressing global change.

Doubly, we believe the problem starts much earlier in education. Within the UK's primary national curriculum, students are only required to identify and name a variety of common wild and garden plants during their early school years with little additional plant ecology or natural history, while no identification skills are taught in secondary education (Table A5). Plant teaching in secondary education is focused on bioenergetics, reproduction, and anatomy with little on plant ecology and no identification skills. In our analysis of the UK School Curriculum, we note few references to exploring plant diversity and ecology, mostly for young children (Table A5). One study of A‐Level biology students in the United Kingdom found only 14% could recognize more than three species of native plants, a trend which matched their teachers' botanical skills (Bebbington, 2005). Elsewhere, a similar experiment in the United States found college students could, on average, only correctly list a single species of wildflower, “weeds,” or grasses when tested on these groups separately (Wagner, 2008). However, there is generally a dearth of studies in this area, with few studies attempting to understand UK students' botanical skills within higher education.

It has been a decade since a student was enrolled in a solely botanic degree in the United Kingdom (Drea, 2011). Of course, the nomenclature of the degree awarded is less important than the education provided: Botany is but a word (Drea, 2011). Throughout their experience of education, biology is predominantly taught by teachers and academics that come from a background of studying animals rather than plants (Colon et al., 2020). Wandersee and Schussler (1999) argue that often even knowledgeable biologist educators may offer only a cursory glance at botany often only in the context of animals. Future to this, opportunities to engage with field botany may be considered expensive, high risk, and logically time‐consuming by institutions and schools (Lambert & Reiss, 2014; Thomas & Munge, 2017). It is this decline in engagement and uptake in teaching students about the fascinating biology of the plant kingdom which is the hidden extinction (Drea, 2011); however, when students are exposed to plant content often their reaction is positive (Barratt, 2011; da Silva et al., 2012). We should seek to deliver a botanical education, one in which the degree awarded is recognized to have a holistic understanding of plants; a degree that centres the values of plants in their broader biochemical, ecological, and social contexts.

According to HESA (The Higher Education Statistics Agency), the total number of graduates from general biology programmes in the United Kingdom between the period 2007 and 2019 was approximately 104,895, while those enrolled in plant science and plant biology programmes accounted for <0.05% of these students (*n* = 565). For every 185 general students of the biological disciplines educated, only a single student of the botanical sciences is produced in the United Kingdom (Table A2). Additionally, we calculated the attrition and recruitment of students to plant science degrees over this period and found that on average there was an average 5.5% gain in student recruitment to these programmes when compared to their first‐year cohort (Table A3). Although there was a wide degree of variation in this, with a wider cohort having a large degree of attrition and recruitment (Table A3). For example, the 2009/10 cohort and the 2012/13 cohorts lost 20% and 39% of their students whilst the 2007/8 and 2008/09 increased in cohort size by 25% (Table A3).

We also note that there may be some overlap with agriculture and forestry courses, while plants, plant processes, and plant products may be a strong focus of these degrees' students are unlikely to develop a strong species literacy within these programmes as they focus on specific subgroups of plants for very specific purposes, not broader plant diversity and ecology knowledge. However, we find little evidence within the literature to confidently access plant awareness in the UK agricultural and forestry education sectors.

To further investigate the plant content of UK biology degrees, we selected all biology degrees and programmes provided by Russell Group universities. We chose Russell Group universities as these are some of the UK's leading research and teaching institutions. With the majority frequently being featured within the world's top 100 universities ranking and dominant in attracting research income (Shattock, 2017). We, therefore, felt that these institutions would therefore provide a valid overview of the teaching biology student receive within the UK higher education sector.

From the latest available module catalogue, we extracted the total number of modules offered across the degree programme and then pulled out modules which contain some degree of plant content as per the module description. We then categorized them into one of several categories. These categories were:

1. Ecology and affiliated life science (implied focus on plants or strong ecological context),
2. Key or central plant focus,
3. Modules with some ID component (a vague nondescript reference to identification i.e., species identification skills), or
4. Modules with clear plant ID component (specifically listed identification of plants).

We additionally pulled out those modules which focused on animal or zoological content for comparison (Table A1). This analysis was reliant on the accuracy of programme module catalogues and their module descriptions and as such may not have captured the entirety of plant content offered to undergraduates.

For example, the University of Leeds offers a BSc (Hons) Biology degree which has a total of 65 modules. Ten of these modules focus on ecology and affiliated life sciences with an implied focus on plants or strong ecological contexts such as *Coastal and Upland Habitats Field Course, Level 3 Field Course (South Africa), Advanced Topics in Evolution*, and *Advanced Topics in Conservation Science*. Six with dedicated animal content such as *Animal Behavior, Organismal Evolution, and Parasitology*. Eight of them had a key or central plant focus such as *Applied Biology and Agriculture, How Plants Work, Sustainable Food Production*, and *Plant Development*. One module with some ID component is *the Mediterranean Ecology Field Course*. And only two modules with a clear plant ID component, *Exploring Whole Organism Biology in the Lab and Field*, and *Urban Ecology and Conservation Field Course*. We excluded Cambridge and Oxford due to their unique degree pathways which did not fit into our analysis framework. There is a degree of uncertainty to this method as the delivery of teaching content may likely change periodically or may not be explicitly stated in module titles or descriptions. We recognize that this method does not take account of teaching on degrees outside of biology programmes and some plant content for modules may not have been included within module descriptions (e.g., many Geography degrees will include some plant‐focused teaching).

Of the 971 modules offered within biology degrees by Russell Group universities in the United Kingdom, we estimate that only 6% (*n* = 63) had a clear plant biology emphasis and only 1% (*n* = 10) had a significant emphasis on plant identification (Table A1). Additionally, the modules offered mostly focus on physiology or agricultural and industrial application of plant science (Table A1). More alarming is the content of plant biology programmes. Modules with a dedicated plant focus contribute only 22% of taught content, and plant identification components are rare, accounting for only 1% of modules (Table A4). For both biology and plant biology programmes, we note a sizable proportion of ecology and associated life science modules; however, often these referred to plants and habitats in generic and nondescript terms, generally alluding to animal and human case studies rather than having a clear plant‐focus (Table A1).

It is often that plants are seen as background characters in the story of an ecosystem, they are simply passive set‐dressing for the lives of the animals which live out their dynamic lives (Balding & Williams, 2016; Yorek et al., 2009). There is a need for people to understand the ecological role, value, and knowledge about species and ecosystems. This concept is known as *species literacy*. Species literacy is more than naming species: It is knowledge not just of an organism's biology, environment, life cycles, and other key ecological details, but also competencies and skills such as observation of species and application of knowledge from multiple plant‐related disciplines (Hooykaas et al., 2019; Magntorn & Helldén, 2005). Integrating concepts such as species literacy into education will enable students to engage with and better value the full spectrum of ecology. We argue that without the integration of identification, plant ecology, and field skills students are limited in their capacity to understand and value the species and processes within these systems.

Often biology textbooks utilize a far greater number of animal examples. One study identified that there were more than double the number of animal examples than plant examples in science textbooks (Schussler et al., 2010), a more recent analysis by Brownlee et al. (2021) indicates this phenomenon has not changed drastically, with animal examples being more predominate across four common introductory biology textbooks (Brownlee et al., 2021).

The above studies highlight the phenomenon of plant blindness. Plant blindness, described by Wandersee and Schussler in 1999, is the inability of a person to perceive plants in their environment, acknowledge their importance in the biosphere or appreciate plants' esthetic and unique biological features. It also refers to the misguided anthropocentric view of plants as inferior to animals (Wandersee & Schussler, 1999). This animal‐focused view, referred to as zoochauvanism, results in diminished plant content within education from primary to university level and globally results in the loss of botanical knowledge, skills, and appreciation (Hershey, 1996).

Plant blindness is a complex and multifaceted phenomenon that encompasses several different aspects related in complex ways. Plant blindness is not only an exclusively sociological and cultural phenomenon but also a multifactorial phenomenon that is affected by evolutionary and cognitive factors (Balding & Williams, 2016). Balas and Momsen (2014) conducted a study on “Attentional blink,” a phenomenon in perception related to visual attention, and the results of the study suggested the visual system may process plants in a manner that may contribute to plant blindness (Balas & Momsen, 2014). They argue that botanists and educators should focus on the design of material that increases plant awareness and directs students in how to compensate for and overcome these inherent perceptual limitations (Balas & Momsen, 2014). These sentiments have been mirrored by others in the field; for example, Balding and Williams further discuss cultural factors in the differing appreciation of flora and fauna (Balding & Williams, 2016).

However, it should be noted that various other terms for plant blindness have been suggested including “plant unawareness” or “plant awareness disparity” (Parsley, 2020). This language places negative connotations on those experiencing these phenomena and various educators have suggested alternative concepts such as fostering “plant awareness” (Bacon et al., 2021).

Many young people are aware of the challenges facing our communities and enthusiastically engage in actions such as climate activism (O'Brien et al., 2018) but are not taught about plant diversity and ecology. To continue to inspire people, we need to teach them that they are part of an ecology, and to do this, a botanically species literate education is essential (Stagg & Donkin, 2013). Compounding this is the occasionally flippant attitude toward the skills of botanists (Manzano, 2021), with some describing elements of botany as a descriptive discipline without scientific rigor (Crisci et al., 2020). To continue to lose plant‐focused education, we lose opportunities for students to engage with the botanical world.

We suggest that, as more botany departments are lost (Sundberg, 2004), plant ecology pared from curricula, and amateur and professional botanists retire (Crisci et al., 2020), we are at a heightened risk of losing collective generations of knowledge.

### 1.2 The global context

This appears to be a global trend. In the United States with the dissolution of botanical institutions, Crisci discusses the current “process of pervasive denigration” of the science (Crisci et al., 2020). More recently, indigenous and local communities' knowledge of landscapes and habitats has been recognized as critical to global conservation goals (Conservation Matters, 2021). One Swiss study of several thousand participants aged between eight and 18 could on average only identify five plants, although this study also noted there was a generally poor ability to recognize species (Lindemann‐Matthies, 2002). Analysis of South African educational texts followed similar trends to other studies, with the authors noting the content taught is likely not sufficient to provide a strong knowledge or skills foundation in the plant sciences and is subsequently unlikely to encourage positive development of values toward plants (Abrie, 2016). Concerningly, recent research revealed potential threats to indigenous knowledge and observed economic development led to reductions in local ethnobotanical knowledge (Saynes‐Vásquez et al., 2016).

The threat of skills debts has also been noted in the Australian plant agricultural sector, where concerns over the small number of agricultural experts to fulfill future food demand have been raised (Merriman, 2012). We suspect there is a ubiquitous loss of identification skills across botanical and environmental sectors; however, we found no studies which explore this.

What is better documented is the decline in taxonomy, which has been noted since the 1970s (Hopkins & Freckleton, 2002; Lee, 2000; Tilling, 1987). Efforts to address these skills gaps have been unsuccessful, with training and recruitment failing (Drew, 2011; Ely et al., 2017). In the United Kingdom, the situation is urgent. A review found that “taxonomy and systematics in the UK is unsatisfactory – in some areas to the point of crisis” (Boxshall & Self, 2011). One of the key concerns of the report was the lack of young individuals entering the workforce with taxonomic skills. There is no evidence that the situation has changed in the last ten years. Beyond the decline of the science of taxonomy, there is the “extinction of experience,” coined three decades ago by Robert Pyle, which describes humanity's ongoing disconnection with nature (Miller, 2005).

Pyle describes disconnection from nature via a “cycle of impoverishment that is initiated by the homogenisation and reduction of local flora and fauna, followed by disaffection and apathy.” Ultimately, this impoverishment is intensified by a “shifting baseline,” progressively decreasing expectations of the quality and function of the natural areas individuals are exposed to (Miller, 2005). It has been suggested that the extinction of experience may be a crucial social‐environmental influence of our time, directly dictating the public support for environmental action and policy (Gaston & Soga, 2020).

Building upon Pyle's and others' work on plant blindness, we propose an additional process by which people have become disengaged from the botanical world. The paring and removal of many botanical‐based skills and subjects from across educational curricula have driven multiple knowledge and skills loss feedback loops, resulting in an *extinction of botanical education*. A cycle that further drives the multiple mechanisms that facilitate and ultimately resulted in widespread plant blindness.

**FIGURE 1:**
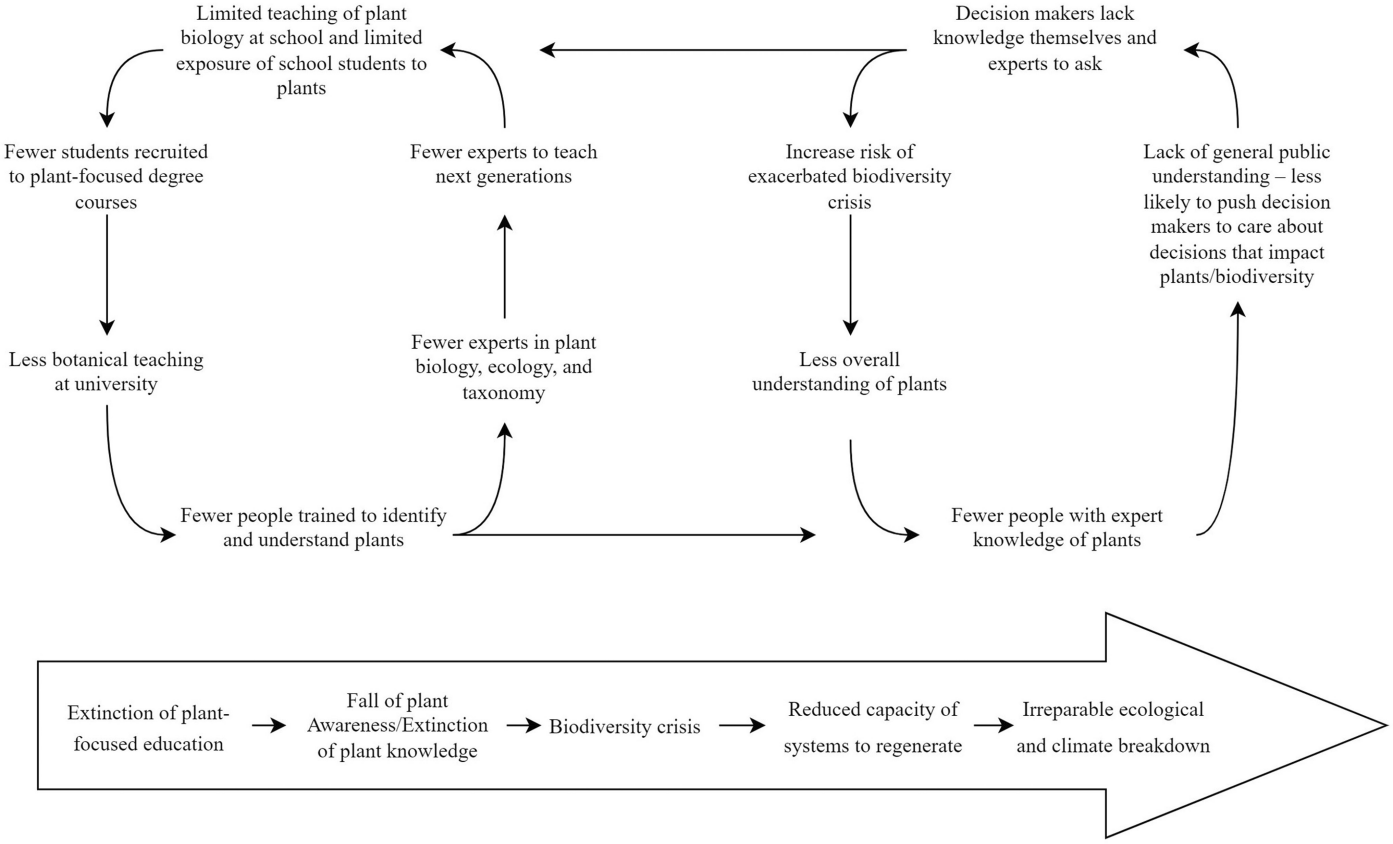
The fall of plant awareness and the extinction of botanical education. The decrease in plant awareness feeds into a cycle of diminishing knowledge of plants at both expert and general knowledge levels that will ultimately increase the risk of the biodiversity crisis and the potential for resilience and restoration in the face of anthropogenic global change

At its core, the extinction of botanical education is comprised of two simple interacting cycles (Figure 1): the fall in plant awareness through a lack of exposure to plants and a loss of knowledge through a diminished demand and provision of botanical education. However, the consequences of these two simple interacting phenomena, if not reversed, may have irreparable and disastrous consequences for our society. Where do the hands of the Doomsday Clock lie for botanical expertise? How many generations of botanists remain before we no longer have the expertise to understand the tipping points of our ecosystems? The longer we allow the cycle to continue, the more difficult it will likely become to halt and reverse it.

We are not the first to recognize this phenomenon of botanical knowledge loss. Multiple papers have discussed the decline of botany as a science (Crisci et al., 2020; Drea, 2011), but often these papers have focused on the threats to biodiversity (Baldini et al., 2021; Prather et al., 2004) without a focus on the broader existential threats.

## 2 THE RISKS OF AN EXTINCTION OF BOTANICAL EDUCATION

### 2.1 Capacity building and defining risk

Capacity building is a system of action developed by the United Nations Development Programme to aid organizations to perform their duties more effectively. It is the process by which individuals are supported via policy and legal frameworks, institutional development, and community participation to develop skills, and knowledge and gain access to resources to enable them to perform efficiently (Moreno et al., 2017) and is a priority within many environmental conventions (Rawat et al., 2008). The Scottish government has acknowledged the workforce needed to effectively implement nature‐based solutions (NatureScot, 2021), which are directly related to plant systems and processes (Amprazis & Papadopoulou, 2020), is currently insufficient in number, skills, and training (NatureScot, 2021). The UK STSC made a similar recommendation in their Nature‐based solutions: rhetoric or reality, report. The report notes the United Kingdom does not currently have the necessary skills to effectively deliver nature‐based solutions at scale, with emphasis on vegetation‐based solutions. This has been acknowledged by the Government but there has been no formal assessment of the skills needed, nor the routes to providing training in the timescales required for a transition over the next decade (UK Gov Science and Technology Select Committee, 2021). These skills deficits are firmly within the botanical realm, covering disciplines from forestry and ecology to peatland restoration (UK Gov Science and Technology Select Committee, 2021).

There have also been calls for urgent action in the British horticultural industry. For example, the Horticulture Matters Campaign supported horticulture and guidance within schools and promoted apprenticeships and horticultural courses in response to a growing need for horticulturalists (Biggs, 2014). The situation highlighted both the need and demand for plant awareness to be on the educational agenda, with one 2014 Royal Horticultural Society survey of 200 businesses finding only 32% could fill listed job vacancies (Biggs, 2014).

This principle can be applied to the extinction of botanical knowledge. The chance of a detrimental event happening is *risk*; there is a definable numeric value to the extent of the anticipated loss and the likelihood of occurrence (Saynes‐Vásquez et al., 2016). The 2010 UK Taxonomy & Systematics Review noted that university training was failing to produce appropriately competent graduates or postgraduates (Boxshall & Self, 2011). There is a push to reverse this shortage: For example, in 2019, the United States reintroduced the so‐called “Botany Bill” in response to alerts from agencies that could not find skilled botanists to deal with invasive plants, wildfire reforestation, and basic land management and fears that through attrition almost half of botanical experts the United States will retire without replacement (Crisci et al., 2020).

The continued decline of botanical knowledge will have far‐reaching impacts on multiple sectors where botanical knowledge is vital. We briefly consider two disciplines as examples and the risks of the extinction of botanical education and plant blindness pose: palaeoclimate research and invasive non‐native species research.

### 2.2 Palaeoclimate reconstructions

Botanical studies have been critical for understanding the evolution and structure of terrestrial ecosystems and the interactions of plants and the atmosphere as well as understanding how plants have responded to previous periods of mass extinction (McElwain & Punyasena, 2007). Studies of stomata helped understand how plants respond to changing atmospheric CO_2_ and how much this has changed over geological time (Woodward, 1987). Studies of plants and palaeobotany are critical for understanding how plants have responded to periods of intense global change in the past and help us to understand and predict how they may respond to changes in the near‐term and far future.

### 2.3 Invasive non‐native species

The cost of invasive species to global economies is staggering with many of the highest impact species including plants (Zenni et al., 2021). In South Africa alone, the damage to fynbos ecosystems is estimated to exceed US$11 billion, with some species such as black wattle (*Acacia mearnsii*) amounting to damages approximate to US$1.4 billion (van Wilgen et al., 2001). The inability to identify invasive species or sounds botanical knowledge continues to exacerbate this problem, may people have poor plant identification skills and are thus unable to recognize invasive non‐native plants, this feeds into reporting, spread or mismanagement(Hester & Cacho, 2017). As such, basic identification skills are increasingly important for the public to know if they have an invasive species growing locally and, particularly, to make informed decisions about plant purchases. Without a well‐equipped, knowledgeable public, effective management of invasive non‐native species, such as essential early detection is infeasible (Vander Zanden et al., 2010). Beaury et al. (2021), recently found that 61% of species listed as invasive in the United States remain for sale, which highlights a significant problem with sound ecological decision‐making even among considerably plant‐engaged people.

Beyond these impacts, there is so much more that comes from our relationship with plants. They inspire a sense of place (Elmendorf, 2008), support our mental and physical health (van den Bosch & Ode Sang, 2017) and many have significant cultural importance around the world (Kandeler & Ullrich, 2009). For example, the utilization of green space during the Covid‐19 pandemic was the focus of a study that found the UK government's decision to enable residents' outdoor recreational activities delivered the equivalent of £1.14 billion in welfare benefits to residents of England (Day, 2020). One study estimated the ecosystem services value of New York City's Central Park to be in the region of $70 million per hectare per year (Sutton & Anderson, 2016), while estimates of annual air pollution removal by the urban forest in the United States are valued at 3.8 billion US dollars (Nowak et al., 2006).

## 3 REVERSING THE EXTINCTION OF BOTANICAL EDUCATION‐PUTTING PLANTS ON THE AGENDA

Botanists and educators have documented the decline in botanical teaching for decades (Crisci et al., 2020; Drea, 2011; Godwin, 1968), but have we produced a clear set of definable and reportable actions and objectives to reverse it?

We suggest that the following research agenda be prioritized to enhance botanical teaching in schools and universities and counter the extinction of botanical education.

- To achieve this, a research baseline from schools and universities to identify the current provisioning of botanical teaching globally is needed.

- To understand current stances and perspectives of pedagogical professionals about the state of botanical education, student engagement, and ethnobotanical knowledge transfer and practice.
- To develop a system for monitoring long‐term trends in botanical knowledge across various demographics and related industries.

- A wider scope of pedagogical methods for enhancing plant identification skills and developing appreciation and value in different demographics and disciplines is needed.

- A review of the literature, skills gaps, and curricula across educational and professional sectors to identify capacity building needs, skills gaps and research opportunities
- Undertake scoping excise to develop QAA content within modules that bridges the gaps that have botanical teaching within applied, plant‐based disciplines such as agriculture and biotechnology, where plants are utilized, but contextual botanical understanding is often poor
- Increase botanical content delivery in a range of subjects for example medicine, veterinary sciences, civil engineering, political science, governance, the arts to grow plant awareness and the value of plants in the higher education community
- To communicate the value of plants should be through public awareness campaigns and various media to educate and engage people from infants to adults

Educational curricula require a profound overhaul to better include the delivery of plant content. It is not necessary to recall esoteric taxonomic language and relationships but rather to elucidate plant stories, survival strategies, and relevance to contemporary society. We argue, plant knowledge can be taught from a variety of perspectives, and all are needed, but not everyone needs all.

Students should not leave school only able to identify two or three plants but should retain the knowledge of the ecological importance of plants to develop a species literacy, particularly the role of plants in solving modern societal challenges. Teachers, many of whom have not had a strong botanical education themselves, having been taught in a zoochauvinist world need to be empowered and supported to gain the skills to teach botany confidently. This zoochauvinist bias is difficult to unlearn and humans have been described as “everything‐but‐mammal blind.” If this bias is not acknowledged within our reversal of this cycle, efficient solutions will not be identified (Clark & May, 2002; Knapp, 2019). Many university students are engaging with ecology, but without strong identification skills, plants will continue to be background players in the stories of an ecosystem. There are institutions whose aims are to actively combat this extinction of botanical education such as The Gatsby Plant Science Summer School (Levesley et al., 2012); however, their focus is mostly on applied plant science rather than a broad botanical education.

We believe the key is to ensure a strong holistic plant narrative that focuses on plants' critical importance to society and global change in curricula that permeates primary through to university education. Framing personal narratives between individuals and plants enable us to increase nature connectedness, which is a better predictor of the pro‐environmental conservation behaviors we wish to facilitate (Richardson et al., 2016). Now may well be the time, a recent paper has identified that over the last decade public interest in plant‐related topics has increased, particularly with the advent of social media influencers, as such there may be a window of opportunity to develop an appreciation of plant science among the wider public, who to some degree are reconnecting with plants (Burke et al., 2022).

Botanists and others in allied disciplines can support these goals and ambitions but, ultimately, change needs to come from those who define policy. Policies must support the science and skills of botany in schools and universities. Hawken notes that fear of a future characterized by environmental degradation has rarely been an effective motivator for people; thus, it is now time to engage with botanical opportunities (Miller, 2005). As such, we must pose the extinction of botanical education not only in terms of financial risk but also in opportunities for positive social change for institutions, policymakers, and funding organizations. Increasing awareness of both the climate and biodiversity crises has led to increased engagement, particularly from younger generations (Wachholz et al., 2014). Botany has a significant role to play in supporting this societal transition and needs to be included in the solution. Increased botanical education is the first step to achieving this, and we should encourage the exploration of the beauty, fascination, and importance of plants from preschool to postgraduate.

## 4 CONCLUSIONS

An invested and knowledgeable public is one equipped to drive environmental policy reform. A plant aware public will only be achieved through education at all levels. The extinction of botanical education will only continue to worsen unless we break the cycle of disconnection from the botanical world.

There is a critical need to address growing skills gaps in the general and botanical sciences and to reengage the wider public with the value of plants before we reach irreversible tipping points of knowledge and biodiversity decline. We must foster environmentally sympathetic attitudes and skills in the wider population and combat this extinction of botanical education, loss of botanical knowledge and loss of technical skills to grow plant awareness. A key component of this is through formal educational routes via the integration of more plant‐focused teaching to connect people with the value of plants. We need profound and comprehensive educational reform to develop students' attitudes and knowledge of plants to enable students to develop the skills and motivation needed to reverse the decades of environmental degradation, neglect of plant value, and support the transition to an ecological and sustainable society.

Plants have significance to every person on the planet, most just do not know it yet.

## AUTHOR CONTRIBUTIONS

**Sebastian Stroud:** Conceptualization (equal); data curation (lead); investigation (lead); project administration (lead); writing – original draft (lead); writing – review and editing (equal). **Mark Fennell:** Conceptualization (equal); writing – original draft (supporting); writing – review and editing (equal). **Jonathan Mitchley:** Conceptualization (equal); writing – original draft (supporting); writing – review and editing (equal). **Susannah Lydon:** Conceptualization (equal); writing – original draft (supporting); writing – review and editing (equal). **Julie Peacock:** Conceptualization (equal); supervision (equal); writing – original draft (supporting); writing – review and editing (equal). **Karen Bacon:** Conceptualization (equal); project administration (supporting); supervision (equal); writing – original draft (equal); writing – review and editing (equal).

## CONFLICT OF INTEREST

The authors state no conflict of interests.

## Supporting information


**Appendix S1** Supporting information

## Data Availability

The data that supports the findings of this study are available in the Supporting Information of this article.

## Appendix

**TABLE A1:** Biological Science course breakdown from 24 Russell group university in the United Kingdom

University	Program title	Total modules offered	Ecology and affiliated life science	Key animal focus	Key plant focus	Some plant ID component	Strong plant ID component
University of Birmingham	BSc Biological Sciences	37	4	2	7	1	1
University of Bristol	Biology (BSc)	39	11	5	7	0	0
Cardiff University	Biological Sciences (BSc)	36	8	1	3	1	0
Durham University	Biological Sciences	45	6	2	1	1	0
University of Edinburgh	Biological Sciences	66	15	13	12	1	1
University of Exeter	BSc Biological Sciences	49	5	3	7	0	0
University of York	BSc (Hons) Biology	36	6	2	2	0	0
Imperial College London	Biological Sciences	44	8	2	3	2	0
University of Leeds	BSC Biology	65	10	6	8	2	2
University of Liverpool	Biological Sciences (BSc)	102	8	0	15	3	0
University of Manchester	BSc Biology	76	8	1	7	1	0
Newcastle University	Biology	39	5	4	7	1	1
University of Nottingham	Biology BSc	50	10	5	10	1	0
Queen Mary University of London	Biology	36	5	0	4	2	0
Queen's University Belfast	Biological Sciences	25	5	1	5	0	0
University of Sheffield	Biology BSc	52	22	6	7	0	2
University of Southampton	Biology (BSc)	67	7	3	9	2	1
University College London	Biological Science BSc	68	9	1	7	0	1
University of Warwick	Biological Sciences BSc	25	4	2	1	0	0
London School of Economics	No comparable program	0	0	0	0	0	0
University of Glasgow	No comparable program	0	0	0	0	0	0
King's College London	No comparable program	0	0	0	0	0	0
Total	19	957	156	59	122	18	9

**TABLE A2:** Exit award for UK undergraduate degree qualifiers in biology and agriculture disciplines

Year	(C1) biology	(C2) botany	(C3) zoology	(C4) genetics	(D4) agriculture	(D5) forestry	(D7) agricultural sciences	Total	Plant science and allies	Plant sci in total (%)	Plant and allied in total (%)	1st year enrollments plant sci	Change plant sci enrollment	% change plant sci enrollment
2007/08	4730	45	895	485	1010	55	70	7360	1180	0.61	16.03	40	−5	−11.11
2008/09	4665	35	925	465	910	65	55	7175	1065	0.49	14.84	40	5	14.29
2009/10	4760	50	970	460	915	80	5	7245	1050	0.69	14.49	50	0	0.00
2010/11	5050	50	1065	420	1000	60	5	7655	1115	0.65	14.57	30	−20	−40.00
2011/12	5170	40	1240	470	1055	75	0	8050	1170	0.50	14.53	40	0	0.00
2012/13	5915	30	1205	480	1120	75	0	8825	1225	0.34	13.88	65	35	116.67
2013/14	6480	45	1490	500	1235	95	0	9845	1375	0.46	13.97	50	5	11.11
2014/15	6030	40	1315	450	1070	60	0	8965	1170	0.45	13.05	50	10	25.00
2015/16	6295	65	1445	510	1055	65	0	9435	1185	0.69	12.56	45	−20	−30.77
2016/17	6630	55	1600	540	1155	55	0	10,035	1265	0.55	12.61	60	5	9.09
2017/18	6920	50	1590	550	1020	35	0	10,165	1105	0.49	10.87	75	25	50.00
2018/19	6840	60	1505	595	1105	25	5	10,140	1195	0.59	11.79	70	10	16.67
Total	69,485	565	15,245	5925	12,650	745	140	104,895	14,100	0.54	13.44	615	50	8.13

*Note*: Data shown are all home/EU/overseas student for the years 2007–2019. Plant science and allies consists of botany, agriculture, forestry, and agricultural sciences. These data are rounded by HESA to the nearest five and as such there is a degree of uncertainty regarding the precise number of students.

**TABLE A3:** Recruitment and attrition from plant science and plant biology degree programmes in the United Kingdom

Academic year	First year cohort	Graduate cohort	Change cohort	Total of cohort
2007/08	40	Na	Na	Na
2008/09	40	Na	Na	Na
2009/10	50	50	10	25.0
2010/11	30	50	10	25.0
2011/12	40	40	−10	−20.0
2012/13	65	30	0	0.0
2013/14	50	45	5	12.5
2014/15	50	40	−25	−38.5
2015/16	45	65	15	30.0
2016/17	60	55	5	10.0
2017/18	Na	50	5	11.1
2018/19	Na	60	0	0.0
Total	470	485	15	5.5

*Note*: Percentage change in graduating students after 3 years. HESA data are rounded to nearest five and as such there is a degree of uncertainly in the accuracy of these data.

**TABLE A4:** Module breakdowns from 11 institutions offering plant science or plant biology degrees in the United Kingdom

University	Program title	Total modules offered	Ecology and affiliated life science	Key plant focus	Some plant ID component	Strong plant ID component
University of Aberdeen	Plant and soil science BSc	47	13	3	2	1
Aberystwyth University	Plant biology BSc	26	3	8	0	4
Canterbury Christ Church University	Plant science	22	1	7	1	0
Edge Hill University	Plant science	30	7	4	0	3
University of Nottingham	Plant science	30	5	16	0	1
University of Sheffield	Plant science	44	10	11	1	1
University of Glasgow	Molecular and Cellular Biology (with Plant Science) BSc (Hons)	25	1	1	0	0
University of Manchester	BSC Plant Science	67	7	2	0	1
University of Bristol	Plant Sciences	39	7	7	0	0
Newcastle University	Applied Plant Science BSc (Hons)	0	6	14	0	2
University of Edinburgh	Biological Sciences (Plant Science) (BSc) C200	66	11	13	1	1
Total		396	70	86	5	14

**TABLE A5:** Current UK national curriculum plant identification and ecology content (Department for Education, 2015)

**Key stage 1 programme of study—years 1 and 2** *Year 1 programme of study* *Pupils should be taught to:* *Identify and name a variety of common wild and garden plants, including deciduous and evergreen trees* *Identify and describe the basic structure of a variety of common flowering plants, including trees* *Year 2 programme of study* *Identify and name a variety of plants and animals in their habitats, including microhabitats* **Lower key stage 2 – years 3 and 4** *Year 3 programme of study* *Identify and describe the functions of different parts of flowering plants: roots, stem/trunk, leaves and flowers* *Year 4 programme of study* *Recognize that living things can be grouped in a variety of ways* *Explore and use classification keys to help group, identify and name a variety of living things in their local and wider environment* *Year 6 programme of study* *Describe how living things are classified into broad groups according to common observable characteristics and based on similarities and differences, including micro‐organisms, plants and animals* *Key stage 3* *Bioenergetics + reproduction and anatomy* *Key stage 4* *Bioenergetics +reproduction and anatomy* **As and A level** *AQA—Populations in ecosystems—investigate the distribution of organisms in a named habitat using randomly placed frame quadrats, or a belt transect* *Cambridge International—use suitable methods, such as frame quadrats, line transects, belt transects and mark‐release‐recapture, to assess the distribution and abundance of organisms in a local area* *OCR—how biodiversity may be considered at different‐levels—practical investigations collecting random and non‐random samples in the field. To include habitat biodiversity (e.g., sand dunes, woodland, meadows, and streams), species biodiversity (species richness and species evenness) and genetic biodiversity (e.g., different breeds within a species)*
